# Severe protein-calorie malnutrition in two brothers due to abuse by starvation

**DOI:** 10.1016/j.rppede.2016.06.002

**Published:** 2016

**Authors:** Marcela Montenegro Braga Barroso, Luiza Martins Salvador, Ulysses Fagundes

**Affiliations:** Universidade Federal de São Paulo (Unifesp), São Paulo, SP, Brazil

**Keywords:** Protein-energy malnutrition, Child abuse, Nutritional deficiencies

## Abstract

**Objective:**

To describe the case of two siblings with severe protein-calorie malnutrition due to abuse by starvation.

**Cases description:**

The two patients were simultaneously referred to the Hospital Municipal, where they were admitted to the Pediatric Gastroenterology clinic of a university hospital for diagnostic investigation of the cause of severe malnutrition and screening tests for Celiac Disease, Cystic Fibrosis and Environmental enteropathy among others. The exams were all normal, and after detailed research on the interactions of this family, we reached the conclusion that the malnutrition was due to abuse by starvation. The children spent approximately two months in the hospital, receiving a high-protein and high-calorie diet, with significant nutritional recovery.

**Comments:**

Abuse by starvation, although rare, should always be considered of as one of the causes of child malnutrition and pediatrician should be aware of the child's development, as well as the family interactions, to prevent more severe nutritional and emotional consequences in the future.

## Resumo

**Objetivo:**

Descrever os casos de dois irmãos acometidos de desnutrição proteico-calórica grave causada por abuso por privação alimentar.

**Descrição dos caso:**

Os dois pacientes vieram simultaneamente encaminhados de hospital municipal, onde estavam internados, ao Ambulatório de Gastrenterologia Pediátrica de um hospital universitário para investigação diagnóstica da causa de desnutrição grave. Foram feitos exames para pesquisa de doença celíaca, fibrose cística e enteropatia ambiental, entre outras. Os exames mostraram-se todos normais e, após investigação detalhada sobre o relacionamento dessa família, chegou-se à conclusão de que a desnutrição tinha como causa o abuso por privação alimentar. As crianças passaram cerca de 2 meses internadas, receberam uma dieta hiperproteica e hipercalórica, com recuperação nutricional significativa.

**Comentários:**

O abuso por privação alimentar, embora raro, deve sempre ser pensado como uma das causas de desnutrição infantil, deve o pediatra estar atento ao desenvolvimento da criança, bem como ao seu relacionamento familiar, para evitar consequências nutricionais e emocionais mais graves no futuro.

## Introduction

Protein-calorie malnutrition in childhood is a worldwide public health problem, especially in countries of low and middle income, being related to more than one third of all deaths of infants and children under five years in these countries.^1^

The United Nations Children's Fund (UNICEF) recognizes environmental, economic and socio-political factors as root and underlying causes of malnutrition, with poverty representing the core of the problem.^2^

Another less common, but extremely serious cause concerns abuse by starvation, when parents or caregivers deliberately fail to feed their children, which can lead to risk of death.^3^

The aim of this study is to describe the cases of two siblings suffering from severe protein-energy malnutrition due to abuse by starvation, which characterizes a type of mistreatment.

## Case description

The two patients were simultaneously referred from the Hospital Municipal de Diadema (HMD), where they were admitted to the Pediatric Gastroenterology Clinic of Escola Paulista de Medicina for diagnostic investigation of the severe protein-calorie malnutrition causes.

The mother reported that her younger son, 4 years and 8 months, was feeling fine at home, when suddenly he went into “respiratory arrest” (*sic*); so she called the Mobile Emergency Service (SAMU) and there was need for resuscitation maneuvers (*sic*). After that, the patient was taken to HMD, where it was decided to admit him, together with his older brother, due to a picture of severe malnutrition.

Since then, children started being followed at our outpatient clinic every 15 days for diagnostic investigation and clinical follow-up.

### Case 1

Male patient, aged 6 years and 11 months, with good weight/height gain up to approximately 2 years old. After that time, there was an evident weight gain deceleration, which apparently occurred without any definite cause. It was also observed that between 2 and 4 years of age there was no record of weight and height, because the patient stopped attending the Basic Health Unit (BHU).

After 4 years of age, these measures were again recorded in the vaccination card, which showed evident weight and growth impairment.

According to the mother's report, the patient had an adequate diet (evaluated by a nutrition team). She denied the occurrence of diarrhea, constipation, abdominal pain and/or distension or any other gastrointestinal symptoms.

Regarding the family history, the patient has a brother aged 4 years and 8 months with a similar picture. The father is healthy and the mother was followed at the Psychiatry Service, used medications, but had no definitive diagnosis. At the first visit, the mother said her name was Maria das Graças; however, in subsequent consultations, we realized that their children called her Ana Paula. The mother was investigated on suspicion of mistreatment and accompanied the children during hospitalization at HMD.

The family lived in a house with basic sanitation, with running water and sewage systems. Family income varied from 1 to 5 minimum wages.

On physical examination, the patient showed a regular general status, extremely emaciated, pale (+/4+), apathetic, with scarce subcutaneous tissue, muscle atrophy of the gluteal region and abdominal distension. Weight=8.5kg (W/A *Z*-score=−6.56) and height=87cm (height/age *Z*-score=−6.21).

The patient had the following laboratory tests: hemoglobin 9.4g/dL; hematocrit 28.9%; leukocytes 2680; platelets 148,000; serum glutamic oxaloacetic transaminase (SGOT) 1.191U/L; serum glutamic pyruvic transaminase (SGPT) 1.043U/L. These changes were attributed to a picture of severe protein-calorie malnutrition.

At the first consultation, the diagnosis of severe protein-calorie malnutrition was characterized and the patient started being investigated for the following causes: celiac disease, cystic fibrosis of the pancreas and environmental enteropathy, among others.

**Figure 1:**
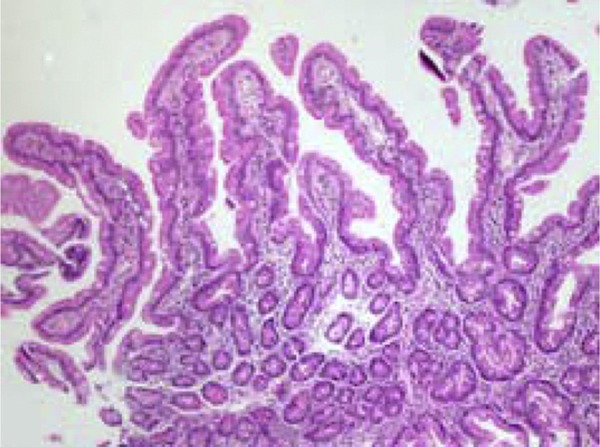
Duodenal biopsy specimens (case 1): mean increase (10×) showing fingerlike villi, preserved crypts, villus:crypt ratio 4:1.

The following laboratory tests were requested: anti-transglutaminase antibody, sweat test (sodium and chloride in sweat) and upper endoscopy with duodenal biopsy. Laboratory tests were negative for the suspected diagnoses and duodenal biopsy disclosed fingerlike villi and celiac disease was ruled out (Fig. 1).

**Figure 2:**
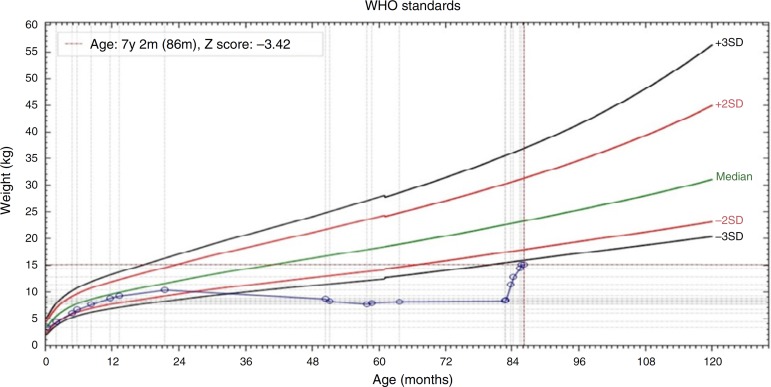
Weight/age ratio chart demonstrating the nutritional recovery in case 1.

**Figure 3:**
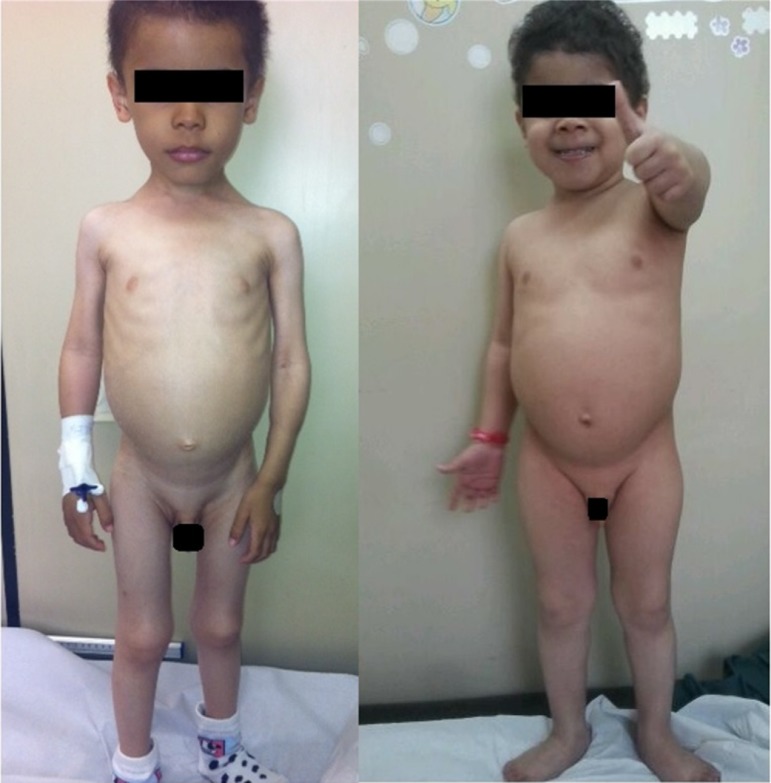
Patient 1 at the first consultation and 2 months later.

The patient was hospitalized for two months at HMD and during the hospitalization period, he received a high-calorie and high-protein diet. The patient showed excellent nutritional recovery (Fig. 2) and considerable improvement in mood and physical activity, with the disappearance of the initial apathy (Fig. 3).

The excellent clinical outcome triggered exclusively by a high-calorie and high-protein diet reinforced the diagnostic suspicion of mistreatment, with subsequent progression to severe protein-calorie malnutrition by starvation. The child was legally removed from their parents and is, together with his brother, in a shelter.

### Case 2

Male patient, aged 4 years and 8 months, born and residing in the municipality of Diadema, was referred to our service from HMD, where he was hospitalized.

The mother reported that the BHU pediatrician started to realize that the patient did not gain weight after 3 years of age. She denied abdominal distension and diarrhea. She reported that the patient ate adequately and, according to the nutrition team assessment, it was not possible to identify any protein-calorie restriction in relation to age.

On physical examination, the child showed regular general status, was very emaciated, weight=7.585kg (weight/age *Z*-score=−5.73) and height=80cm (height/age *Z*-score=−6.35).

**Figure 4:**
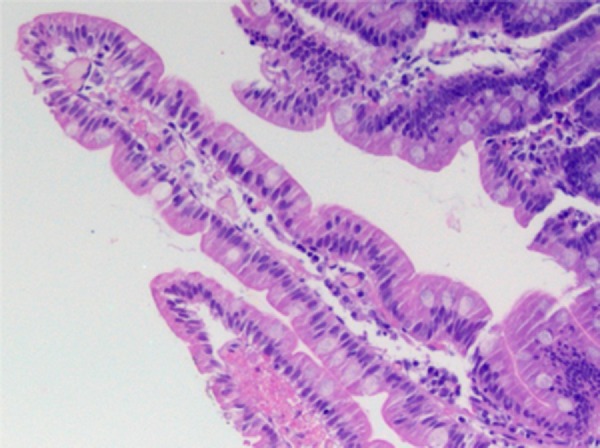
Duodenal biopsy specimen (case 2): high magnification (100×), fingerlike villi, cylindrical epithelium with nucleus in basal position, preserved basal membrane, goblet cells present along the villi, mild lymphoplasmacytic infiltrate in the lamina propria.

At the first consultation, the diagnosis of severe protein-calorie malnutrition was made and the patient started to be investigated for the same causes of malabsorptive diseases that his brother was undergoing, with negative test results. The duodenal biopsy disclosed fingerlike villi and celiac disease was ruled out (Fig. 4).

**Figure 5:**
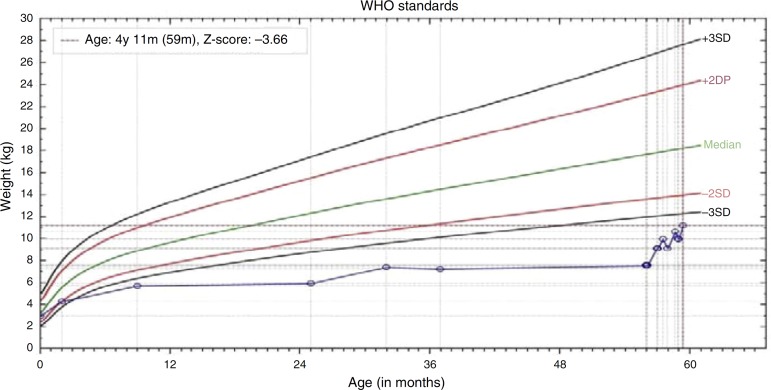
Weight/age ratio chart demonstrating the nutritional recovery in case 2.

**Figure 6:**
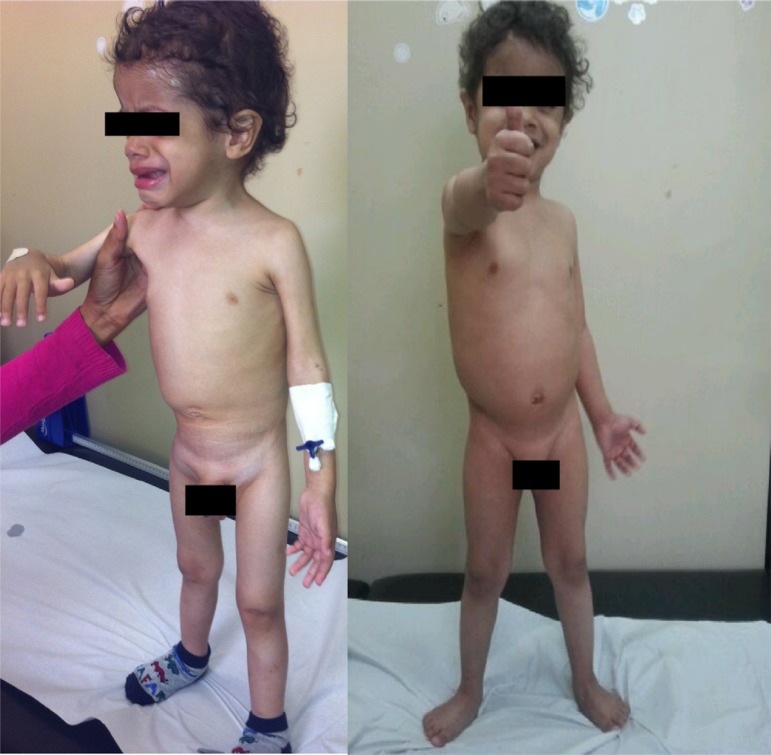
Patient 2 at the first consultation and 2 months later.

The patient remained hospitalized for two months at HMD with his brother, receiving a high calorie and high-protein diet, showing excellent nutritional recovery (Figs. 5 and 6), which reinforced the diagnostic suspicion of abuse.

During one of the visits to our clinic, when the children were already living in the shelter, we asked the social worker about the fact that her mother sometimes said her name was Maria das Graças and, sometimes, Ana Paula. The social worker informed us that up until the older brother was aged 2 and a half years, the children and their parents lived near the rest of the relatives; however, they moved to an unknown destination and lost contact with the family. In this new home, the patient's mother told the neighbors her name was Ana Paula.

Regarding the children's father, he said that he worked all day and did not realize that the mother did not feed children adequately. He was considered an accomplice and the children were kept away from him.

## Discussion

Malnutrition caused by family neglect, which is characterized by a non-deliberate failure to meet the child's needs, can occur due to ignorance, low socioeconomic status or even dietary beliefs.^3^^,^ 4

On the other hand, abuse by starvation, the cause of malnutrition in our two patients, differs from neglect by the fact that it is characterized as deliberate or malicious failure to meet the child's needs.^3^

Nancy et al.,^4^ in 2005, described 12 malnourished children victims of abuse by starvation in Texas, USA. Similarly to the patients in our study, the children were investigated for organic diseases, which were ruled out. These children had, regarding their history, some points in common to that of our two patients, namely: (1) the mother reported that took the children to the pediatrician with some frequency, although there was no recent record of medical consultations; (2) the mother of one of the children went to the health service because the child “was not breathing”, as in the case of our younger patient; (3) the children were also excluded from social life, and in our cases, had no contact with the rest of the family for about two years.

Regarding the fathers' role, Krieger,^5^ in 1974, published 10 cases of children subjected to food deprivation by the mothers, in which the husbands claimed they did not know that the children were subjected to this situation as they spent the day at work and did not realize it, exactly as it happened with the father of the children in our study.

In the national literature, although there are many articles about child abuse, there are few reports on malnutrition caused by abuse due to starvation. Nudelmann and Halpern,^6^ in 2011, published a cross-sectional study carried out in Rio Grande do Sul, in which they evaluated the role of life events in mothers of malnourished children and came to the conclusion that in addition to poor social and economic conditions, these mothers had higher rates of depression, as well as a higher prevalence of abuse during childhood and concluded that malnutrition had a multifactorial origin.

A differential diagnosis that also deserves to be discussed is Munchausen syndrome by proxy, corresponding to a form of child abuse caused by a perpetrator with a psychiatric disorder that exacerbates, falsifies or promotes clinical histories, laboratory evidence, can cause physical injuries and induce hospitalizations for unnecessary therapeutic and diagnostic procedures.^7^

In the cases discussed in this article, the mother, who had a psychiatric disorder, told the emergency service that her child had a “respiratory arrest”, a fact that was not confirmed by the team who treated the child.

A noteworthy aspect concerns the morphological findings in the small intestine at optical microscopy in both patients, considering that the absence of severe injuries could constitute an apparent paradox. However, the description of morphological alterations of the small intestine mucosa in most cases of severe protein-calorie malnutrition is accompanied by environmental enteropathy.^8^^,^ 9 In such circumstances, as previously demonstrated, at least approximately 65% of individuals, even without a complaint of diarrhea, would have an overgrowth of colonic flora in the small intestine lumen.^10^ It is known that the colonization of the small intestine by colonic flora causes malabsorption of dietary nutrients and severe injuries to the small intestine mucosa, due to the 7 alpha-dehydroxylation and deconjugation of primary bile salts, which are converted into secondary bile salts and deconjugated, being very harmful to the jejunal mucosa.^11^ However, this was not the case of our patients, as it was adequately established by the social workers that they lived in a brick house that had water and sewage treatment and, therefore, there were no environmental contamination conditions that could lead to bacterial overgrowth. Moreover, after all relevant laboratory research, it was definitely characterized that the severe protein-calorie malnutrition was exclusively due to deliberate starvation. Experimental studies with rats with severe protein-calorie malnutrition in the absence of triggering factors of bacterial overgrowth showed that the morphology of the small intestine did not suffer any alterations and the villi were fully preserved.

Our patients, in spite of the severe protein-calorie malnutrition by starvation, had intestinal villi perfectly compatible with normality; the inflammatory infiltrate of the lamina propria was discreet and the villus/crypt ratio was 4/1. Interestingly, our patients, as observed in the experimental study of malnourished rats regarding the increased absorptive function, when given a high-protein and high calorie diet, showed rapid nutritional recovery within a short period of time, which proves that their digestive-absorptive functions were kept intact despite the prolonged period of food deprivation. These findings demonstrate for the first time in humans, to the best of our knowledge, that severe protein-calorie malnutrition is not primarily the cause of severe disorders of the digestive-absorptive function: the occurrence of an external factor, such as the environmental contamination, is necessary to trigger all the pathophysiological and symptomatic process described in environmental enteropathy.

Regarding the prevalence of bacterial overgrowth associated with environmental enteropathy in Brazil, some studies have been carried out with the lactulose hydrogen breath test. A study involving 83 schoolchildren living in the rural, urban areas and a slum in a municipality in the countryside of São Paulo disclosed bacterial overgrowth in 7.2% of the assessed children.^12^ In this study, the proportion of bacterial overgrowth in children living in a slum (18.2%) was statistically higher than that of children who did not live in a slum.

Protein-calorie malnutrition can be caused by organic diseases, poor social condition, neglect and abuse by starvation. The latter cause is rare, with few reports in the literature, but it has severe consequences, both nutritional and psychological, for the affected child. The pediatrician plays a key role in the early detection of these cases and should always be alert to monitor the child's weight/height development, as well as the family relationships.
